# Changes in psychiatric emergencies during COVID-19 pandemic lockdown in El Bierzo (Spain)

**DOI:** 10.1192/j.eurpsy.2022.658

**Published:** 2022-09-01

**Authors:** Y. Zapico-Merayo, J.M. Pelayo-Terán, S. Vega-García, M.E. Garcia Llamas, R. Landera-Rodríguez, A. Espandian

**Affiliations:** Hospital El Bierzo. GASBI. SACYL, Psiquiatría Y Salud Mental, Ponferrada, Spain

**Keywords:** incidence, Suicide, emergencies, Covid-19

## Abstract

**Introduction:**

The interest have focused in the effects of COVID-19 in mental symptoms. However, the pandemic and restrictions such as the lockdown decreed in Spain limited access to resources and lead to a change in assistance organization.

**Objectives:**

to compare the incidence and characteristics of psychiatric emergencies during the Spanish 2020 Lockdown with the same period in 2019

**Methods:**

All the emergencies attended the the emergency room (Hospital El Bierzo) From 01/02/2019 to 30/06/2019 and those from 01/02/2020 to 30/06/2020 were analysed by two senior psychiatrists. Cases were selected if attended by any psychiatric reason. The cases were evalueated identifying ICD-10 diagnosis (according to clinical records and best criteria matching), sociodemographics, factors associated to the emergency and resolution.

**Results:**

23360 cases were attended in 2019 (799 psychiatric), 14907 (578) in 2020. That means a 36.19% of reduction in general emergencies and 27.66% in psychiatric emergencies (psychiatric emergencies proportion increased form 3.42% to 4.03%). The reduction started the week just before the lockdown declaration, minimal records coincided with the highest COVID-19 incidence and the recovery starts in early june for psychiatric and late June for general emergencies (figure 1). A decrease of 62.79% of anxiety cases and 45.9% of depresion was observed with no incresaes in any diagnosis. A slight increase in suicide attempts (two cases) was observed.

**Figure:**
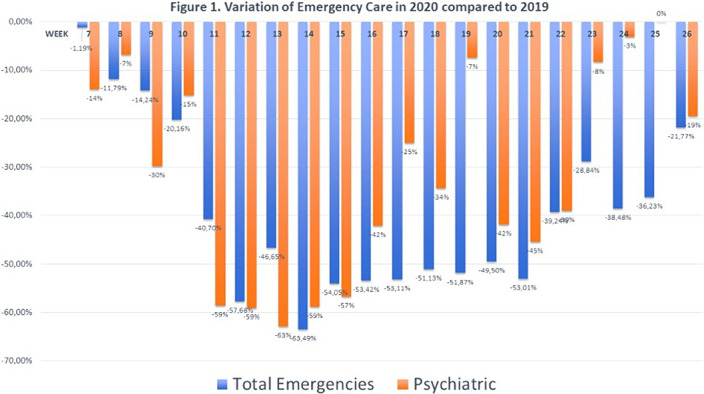

**Conclusions:**

The lockdown seem to decrease psychiatric emergency care. Only suicidability was maintained/increased during the period. Psychiatry services must be aware of the risk of unattended inicidence that may cause an increase of cases after the lockdown.

**Disclosure:**

No significant relationships.

